# The COVID-19 pandemic and mental health outcomes – A cross-sectional study among health care workers in Coastal South India

**DOI:** 10.12688/f1000research.111193.1

**Published:** 2022-06-20

**Authors:** Rekha T, Nithin Kumar, Kausthubh Hegde, Bhaskaran Unnikrishnan, Prasanna Mithra, Ramesh Holla, Darshan Bhagawan

**Affiliations:** 1 Department of Community Medicine, Kasturba Medical College, Mangalore, Manipal Academy of Higher Education, Manipal, India

**Keywords:** Mental Health, Pandemic, Health personnel, Depression, Patient Health Questionnaire, anxiety

## Abstract

**Background: **Frontline health care workers (HCWs) are at increased risk of developing unfavourable mental health outcomes and burnout, especially during the COVID-19 pandemic. Recognizing the early warning signs of mental distress is very important to ensure the provision of quality patient care.

**Methods: **In this facility-based cross-sectional study, HCWs of the teaching hospitals affiliated to Kasturba Medical College, Mangalore were assessed regarding their mental health status using a semi-structured questionnaire. All doctors and nurses who were willing to participate from these teaching hospitals were included in the study. Data was collected over a period of four months (1 ^st^ March -30 ^th^ June 2021) till the required sample size was reached and analysed using IBM SPSS and expressed using mean (standard deviation), median (interquartile range), and proportions. Univariate analysis was done to identify the factors associated with mental health outcomes among the HCWs and the corresponding unadjusted odds ratio and 95% confidence interval were reported.

**Results: **A total of 245 HCWs [52.2% (n=128) doctors and 47.8% (n=117) nurses] were included in our study. The proportion of participants with depressive symptoms, anxiety, and insomnia assessed using PHQ-9, GAD-7, and ISI-7 scales were 49% (n=119), 38% (n=93), and 42% (n=102) respectively. Depression, anxiety, and insomnia were more likely to be experienced by HCWs aged > 27 years, females, and involved in COVID-19 patient care. (p>0.05)

**Conclusions: ** Our findings that 38% of the examined HCWs had clinically relevant anxiety symptoms and 49% had clinically relevant depression symptoms draws attention to the importance of systematically tracking the mental health of HCWs during this ongoing pandemic. HCWs should monitor their stress reactions and seek appropriate help both on a personal and professional level. Appropriate workplace interventions including psychological support should be provided to HCWs, to ensure provision of uncompromised quality patient care.

## Introduction

The coronavirus disease of 2019 (COVID-19), first identified in Wuhan, China spread rapidly, crossing geographical boundaries and infecting millions of people. The World Health Organization (WHO) acknowledged this outbreak to be of immediate public health concern and declared it a pandemic on March 11, 2020. Now, after two years of outbreaks and large-scale vaccination of the susceptible population, there is still no sign of end of the pandemic. As of 25 ^th^ February 2022, WHO estimates close to 430 million confirmed cases and around 59 million deaths due to the severe acute respiratory syndrome coronavirus 2 (SARS-COV2) virus worldwide. ^1^ India, one of the major contributors to the global tally of COVID-19 cases has reported around 42 million confirmed cases and 0.52 million deaths. ^1^^,^^2^ Countries around the world imposed a nationwide lockdown for curbing the rapid spread of COVID-19 infections, which was modified to region-specific restriction of movement of people. The social isolation and loss of livelihood brought about by frequent lockdown had a huge impact on the physical and mental wellbeing of the general population.

The frontline health care workers (HCWs) are at increased risk of developing unfavourable mental health outcomes and burnout. During the pandemic, the HCWs must work relentlessly and for extended hours, attending to huge caseloads and unforeseen medical complications which compounds the mental distress arising out of constant fear of acquiring infection. In India, HCWs involved in COVID-19 care were marginalized and stigmatized. HCWs in many parts of India had to perform their duties under the constant threat of aggression and violence from patient caretakers which heightened their mental distress. Refusal of entry to apartments and residences, resistance to the burial of dead bodies of HCWs, ^3^^,^^4^ and abuse of the doctors involved in screening and contact tracing were reported from different parts of the country. ^5^

Recognizing the early warning signs of mental distress is important in any population, more so among HCWs. Unfavorable mental health outcomes like anxiety, depression, insomnia, psychological distress, and burnout can affect their health, and compromise the patient safety and the quality of care provided. ^6^ Adequate interventions and coping strategies can be implemented if the mental health status of HCWs is routinely evaluated. With this background and in the context of the current COVID-19 pandemic in the district, the study was carried out to assess the mental health outcomes among the HCWs in Mangalore and the factors associated with them.

## Methods

### Ethical approval

The Institutional Ethics Committee of Kasturba Medical College, Mangalore approved the study protocol. (IEC KMC MLR 05-2020/164). Electronic written informed consent was obtained from all participants on the google form. Only consenting participants were able to access the online questionnaire.

### Study area

The study was conducted in the coastal city of Mangalore, belonging to the District of Dakshina Kannada in the Southern part of India. A major commercial and educational hub, an ivory town of hospitals and medical colleges, Mangalore enjoys a high health care index ^6^ catering to patients not only from the adjoining districts, but also from the neighbouring state of Kerala.

Dakshina Kannada is among the top five highly affected districts in the State of Karnataka during the ongoing COVID-19 pandemic with a total of 3.9 million cases reported to date ^7^ with Mangalore being the major contributor to the daily tally of cases.

### Participants

This facility-based cross-sectional study was carried out among the health care workers (HCWs) – doctors and nurses of the teaching hospitals affiliated to Kasturba Medical College, Mangalore.

A total of 245 HCWs were included in the study to assess their mental health outcomes during the COVID-19 pandemic. The sample size was calculated using the formulae for cross-sectional study ^8^ design: N=4pq/d ^2^. It was calculated considering the proportion of HCWs experiencing depressive symptoms to be 50.4% (11), absolute precision of 7%, 80% power, 95% confidence interval, and a non-response error of 20%. Applying the population proportion to size strategy, a total of 128 doctors and 117 nurses were included in the study using non-probability (convenience) sampling method. The doctors and nurses who were willing to participate, were included in the study till the required sample size was reached.

### Data collection instruments

The study was conducted during the second wave of COVID-19 from 1 ^st^ March 2021 to 30 ^th^ June 2021. The information related to study variables was collected using a semi-structured questionnaire in English which had the following sections:

• Section A: General participant information
  This section of the questionnaire included age, gender, designation, specialty, work experience, whether involved in COVID-19 care, and other personal details.
• Section B: Patient Health Questionnaire (PHQ-9) ^10^ - assesses the presence of depression-related symptoms. PHQ-9 consists of nine statements reflecting the participants’ state of mind and was scored on a three-point Likert-type scale from “0” (not at all) to “3” (nearly every day). The total score ranges from 0 to 27.
• Section C: Generalised Anxiety Disorder (GAD-7) scale ^11^ - assesses anxiety among the participants. GAD-7 consists of seven statements assessing the level of anxiousness in a participant like feeling nervous, anxious, or on edge and worrying too much about different things. The statements are rated on a four-point Likert-type scale (0 = not at all to 3 = nearly every day and the scores ranged from 0 to 21 with higher scores indicating more severe GAD symptoms.
• Section D: Insomnia Severity Index (ISI) ^12^^,^^13^ to assess the presence of insomnia.
  The ISI consists of seven-items assessing the level of insomnia across dimensions like severity of sleep onset, sleep maintenance, and early morning awakening problems, sleep dissatisfaction, interference of sleep difficulties with daytime functioning, noticeability of sleep problems by others, and distress caused by the sleep difficulties. All the items are rated using a five-point Likert scale (0 = no problem; 4 = very severe problem), yielding a total score ranging from 0 to 28.
  The questionnaire was pilot tested and validated for the content. Pilot Testing - The pilot testing was carried out in the month of February 2021 among 30 HCWs (15 doctors and 15 nurses). The participants were selected randomly using non-probability sampling. These participants were excluded from the main study. The pilot was done to evaluate the feasibility of an online survey and to finalize the questionnaire. Based on the pilot testing, questions on participants specialty, department to which they belong, and teaching experience in medical college was removed to make section A uniform for both doctors and nurses. No changes were made to Section B, section C, and section D, since they were standard questionnaires and already pre-validated.

***Data collection***

After obtaining the requisite permission from the head of the institution and concerned authorities of the hospitals, a list of doctors and nurses along with their phone numbers and email IDs working in the affiliated hospitals was obtained from the Human Resource department.

To limit the personal contact with the study participants due to the prevailing pandemic, the questionnaire was prepared in Google forms ( https://www.google.co.uk/forms/) and the link was sent to the participants *via* WhatsApp or email. The Google form questionnaire was pre-tested and validated.

The information sheet and consent form were included in the Google form questionnaire. Electronic consent was obtained from each respondent on the first page of the Google form questionnaire. Only the consenting participants were able to access the questionnaire and fill out their responses. We did not include any personal identifiers in the questionnaire to ensure confidentiality of the participants. The link for the survey was circulated till the required sample size of 245 was reached and the final spreadsheet downloaded

### Data management and analysis

The collected data was extracted as a spreadsheet from Google drive and analysed using IBM SPSS (Statistical Package for Social Sciences) Statistics for Windows Version 25.0. Armonk, NY: IBM Corp). The data is expressed using mean (standard deviation), median (interquartile range), and proportions.

The interpretation of the scales used to assess the various mental health outcomes among the participants is as follows:

• General Anxiety Disorder - 7: Normal (0-4), Mild (5-9), Moderate (10-14), and severe (15-21) anxiety.
• Patient Health Questionnaire - 9: Normal (0-4), mild (5-9), moderate (10-14), and severe (15-27) depression.
• Insomnia Severity Index - 7: Normal (0-7), Subthreshold (8-14), Moderate (15-21), and Severe (22-28) insomnia.

The cutoff scores were used for detecting symptoms of major depression, anxiety, and insomnia, Participants were categorized to have severe symptoms if they had scored greater than the cutoff threshold.

For comparison across the groups, (gender, type of HCWs, involved in COVID-19 care) the Mann-Whitney U test was used and a ‘p’ value of <0.05 was considered statistically significant. Univariate analysis was carried out to identify the factors associated with mental health outcomes like presence of depression, anxiety, and insomnia among the HCWs and the corresponding unadjusted odds ratio and 95% confidence interval were reported.

## Results

A total of 245 HCWs, which included 52.2% (n=128) doctors and 47.8% (n=117) nurses were assessed about their mental health status in our study. The mean age of the doctors was 29.7 (±7.9) years, while that of nurses was 26.3 (± 6.2) years. A higher proportion of participants (n=66, 51.6%) among doctors were females. Majority (n=87, 68%) of the doctors and 47% (n=55) of the nurses were involved in the direct care of COVID-19 patients.

The mental health status of the study participants is depicted in Table 1. The proportion of participants with depressive symptoms, anxiety, and insomnia assessed using PHQ-9, GAD-7 and ISI-7 scales were 49% (n=119), 38% (n=93) and 42% (n=102) respectively. The proportion of depression was higher among doctors (51.5%) compared to nurses (45.3%). The anxiety-related symptoms were similar among doctors and nurses (doctors 39.0% vs. 36.7% in nurses). A higher proportion of nurses experienced insomnia related symptoms compared to doctors (45.2% vs. 38.2%) (P >0.05).

**Table 1.** Mental health outcomes among the healthcare workers (HCW) (N=245).

Mental health outcomes	Category of HCW			Chi-square test (p value)
	Doctors (N=128) n (%)	Nurses (N=117) n (%)	Total (N=245) n (%)	
**Level of depression (PHQ-9)**				
Normal (0-4)	62(48.4)	64 (54.7)	126 (51.4)	1.630 (0.661)
Mild (5-9)	37 (29.0)	33 (28.2)	070 (28.6)	
Moderate (10-14)	15 (11.7)	09 (07.7)	024 (09.8)	
Severe (15-27)	14 (10.9)	11 (09.4)	025 (10.2)	
**Level of anxiety (GAD-7)**				
Normal (0-4)	78 (60.9)	74 (63.2)	152 (62.0)	5.131 * (0.159)
Mild (5-9)	27 (21.1)	28 (23.9)	055 (22.4)	
Moderate (10-14)	15 (11.7)	14 (12.0)	029 (11.8)	
Severe (15-27)	08 (06.3)	01 (00.9)	009 (03.7)	
**Level of insomnia (ISI-7)**				
Normal (0-7)	79 (61.7)	64 (54.7)	143 (58.4)	2.550 * (0.451)
Sub threshold (8-14)	36 (28.1)	38 (32.5)	74 (30.2)	
Moderate (15-21)	12 (09.4)	15 (12.8)	27 (11.0)	
Severe (22-28)	01 (00.8)	-	01 (00.4)	

* Fisher exact test.

Comparison of mental health outcome scores as assessed by the various scales is shown in Table 2. The median (IQR) scores for depression (PHQ-9), anxiety (GAD-7), and insomnia (ISI-7) for all the participants were 4.0 (1.0-8.0), 3.0 (0.5-7.0), and 6 (2-10) respectively. The median PHQ-9 and GAD-7 scores were higher among doctors compared to nurses, while nurses had a higher ISI-7 median score. However, no significant difference in mental health scores was observed across categories of HCWs, gender of the participants, and involvement in COVID-19 patients’ care (P>0.05).

**Table 2.** Comparison of mental health outcome scores among health care workers (HCW) (N=245).

Scale	Total participants	Doctors	Nurses	p value *	Male	Female	p value *	Involved in COVID-19 care		P value *
								Yes	No	
	Median (IQR)	Median (IQR)	Median (IQR)		Median (IQR)	Median (IQR)		Median (IQR)	Median (IQR)	
**PHQ-9, depression symptoms**	4 (1-8)	6.2 (2-9)	5.4 (1-8)	0.200	5.8 (1-8)	5.7 (2-9)	0.604	6.2 (1-9)	5.2 (1-8)	0.320
**GAD-7, anxiety symptoms**	3 (0.5-7)	5.0 (1-8)	3.7 (0-5.5)	0.176	4.3 (0-7)	4.5 (1-7)	0.727	4.9 (0.8-7)	4.1 (0-7)	0.495
**ISI, insomnia symptoms**	6 (2-10)	6.6 (2-10)	7.4 (2-10)	0.645	7.2 (2-10)	6.0 (2-10)	0.406	6.8 (2-10)	7.3 (2-10)	0.837

* Mann-Whitney U test.

The risk factors for developing mental health outcomes – depression, anxiety, and insomnia among HCWs is shown in Table 3.

**Table 3.** Univariate analysis showing risk factors for mental health outcomes- depression, insomnia, and anxiety among health care workers (HCW) (N=245).

	Experiencing symptoms of depression		Odds Ratio (95% CI)	P value	Experiencing symptoms of insomnia		Odds Ratio (95% CI)	P value	Experiencing symptoms of anxiety		Odds Ratio (95% CI)	P value
	Yes (N=119)	No (N=126)			Yes (N=102)	No (N=143)			Yes (N=93)	No (N=152)		
	n (%)	n (%)			n (%)	n (%)			n (%)	n (%)		
**Age group (years)**												
≤27	83 (69.7)	83 (65.9)	**1**	**0.521**	75 (73.5)	91(63.6)	**1**	**0.104**	69 (74.2)	97 (63.8)	**1**	**0.093**
>27	36 (30.3)	43 (34.1)	**1.19 (0.70-2.05)**		27 (26.5)	52 (36.4)	**1.59 (0.91-2.79)**		24 (25.8)	55 (36.2)	**1.63 (0.92-2.91)**	
**Gender**												
Male	88 (73.9)	94 (74.6)	**1**	**0.907**	25 (24.5)	105 (73.4)	**1**	**0.000001**	22 (23.7)	041 (27.0)	**1**	**0.571**
Female	31 (26.1)	32 (25.4)	**1.03 (0.58-1.84)**		77 (75.5)	038 (26.6)	**0.12 (0.06-0.21)**		71 (76.3)	111 (73.0)	**0.84 (0.46-1.52)**	
**Health care worker**												
Doctor	66 (55.5)	62 (49.2)	**1**	**0.960**	49 (48.0)	79 (55.2)	**1**	**0.269**	50 (53.8)	78 (51.3)	**1**	**0.712**
Nurse	53 (44.5)	64 (50.8)	**0.78 (0.47-1.29)**		53 (52.0)	64 (44.8)	**0.75 (0.44-1.25)**		43 (46.2)	74 (48.7)	**1.10 (0.66-1.85)**	
**Work Experience (years)**												
≤10	98 (82.4)	88 (69.8)	**1**	**0.331**	85 (83.3)	101(70.6)	**1**	**0.021**	75 (80.6)	111(73.0)	**1**	**0.175**
>10	21 (17.6)	38 (30.2)	**0.49 (0.27-0.90)**		17 (16.7)	042 (29.4)	**2.08 (1.10-3.92)**		18 (19.4)	041 (27.0)	**1.53 (0.82-2.92)**	
**Involvement in COVID care**												
Yes	72 (60.5)	70 (55.6)	**1.22 (0.73-2.04)**	**0.433**	60 (58.8)	82 (57.3)	**0.94 (0.56-1.57)**	**0.819**	54 (58.1)	88 (57.9)	**1.0 (0.58-1.67)**	**0.979**
No	47 (39.5)	56 (44.4)	**1**		42 (41.2)	61 (42.7)	**1**		39 (41.9)	64 (42.1)	**1**	

Depression was more likely to be experienced by HCWs aged >27 years (OR,1.19; 95% CI, 0.70-2.05), female HCWs (OR,1.03; 95% CI, 0.58-1.84), and HCWs involved in COVID-19 care (OR 1.22;95% CI,0.73-2.04). Insomnia was more likely to be experienced by HCWs aged >27 years (OR,1.59; 95% CI, 0.91-2.79) and HCWs with work experience of more than 10 years (OR,2.08; 95% CI, 1.10-3.92). Anxiety was more likely to be experienced by HCWs aged >27 years (OR,1.63; 95% CI, 0.92-2.91), nurses (OR,1.10; 95% CI, 0.66-1.85), and HCWs with work experience of more than 10 years (OR,1.53; 95% CI, 0.82-2.92). However, none of these factors was significantly associated with mental health outcomes (P>0.05), except HCWs with work experience of more than 10 years, which was significantly associated with experiencing insomnia symptoms (P<0.05).

## Discussion

Health-care workers (HCWs) have been at the forefront since the beginning of the pandemic providing uncompromising care to those infected with COVID-19. However, the extended period of duty hours and the constant fear of getting infected or spreading the infection to family members has negatively impacted their physical and mental health. Unfavourable mental health outcomes – depression, insomnia and anxiety were also reported among our study participants.

The prevalence of depression was 51.5% in our study. Anxiety was reported among 38%, while insomnia was present in 42% of the participants. The prevalence of mental health outcomes in our study is high compared to a similar study from another part of India where depression was reported among 47.4% of the HCWs, while anxiety and insomnia were seen among 29.0% and 32.2% of the participants respectively. ^14^ Similar studies conducted among the HCWs during the pandemic from different parts of the world have reported a prevalence of depression ranging from 8.9% to 77.2%, ^15^^–^^25^ anxiety 14.5% to 88%, ^5^^,^^15^^–^^18^^,^^20^^–^^23^^,^^25^^,^^27^^–^^30^ and insomnia 8.3% to 85.4%. ^5^^,^^16^^,^^17^^,^^19^^,^^20^^–^^30^

Many factors can contribute to unfavourable mental health outcomes among HCWs. The long duty hours and overflowing outpatient departments (OPDs), along with acute shortage of trained staff and personal protective equipment (PPE), fear of contracting the infection and spreading the disease to their family members, and continuous performance evaluation results in psychological distress in most HCWs, ultimately leading to burnout. HCWs are also faced with several decision-making dilemmas during a pandemic including allocation of resources, care for a severely ill/dying patient, and aligning patient needs with those of family members further resulting in moral distress. All this is compounded by a prolonged period of separation from family members or a lack of any other form of support system. ^31^^,^^32^

Studies from different parts of the world have reported a variety of factors contributing to unfavourable mental health outcomes. A study from the Eastern Mediterranean region reported that the presence of a pre-existing mental illness, being isolated for COVID-19, and having children was significantly associated with experiencing depressive symptoms ^23^ while insomnia was significantly associated with HCWs working in an isolation unit in a study in China. ^21^ The absence of psychological support at the workplace as a factor for experiencing poor mental outcomes was reported in studies conducted in China and Albania, ^21^^,^^24^ while fear of getting infected and transmitting COVID-19 was associated with experiencing depressive symptoms among HCWs in Switzerland and China. ^20^^,^^21^

The frontline HCWs being directly involved in the examination, diagnosis, and treatment of COVID-19 makes them more vulnerable to contracting the infection. The constant fear of being at risk of getting infected may lead to psychological distress and burnout among them. Several studies have reported that the HCWs involved in direct care for COVID-19 patients were found to have unfavourable mental health outcomes. ^9^^,^^16^^,^^21^^,^^24^

In our study, unfavourable mental health outcomes – depression, anxiety and insomnia were observed among participants >27 years of age, nurses, and HCWs having work experience of more than 10 years. Similar observations were reported in various studies among HCWs around the world. Nurses were found to have a higher risk of experiencing poor mental outcomes in studies conducted in Asia and Africa ^9^^,^^14^^,^^17^^,^^29^ whereas another study from Africa reported male HCWs and physicians to be more at risk for experiencing distress. ^24^^,^^25^ In general, female HCWS were found to be more vulnerable to experiencing depression and insomnia related symptoms. ^9^^,^^14^^,^^17^^,^^19^^,^^20^^,^^28^^,^^29^ Younger HCWs in the age group between 21-30 years were also found to experience unfavourable mental health outcomes in several studies. ^16^^,^^25^^,^^29^^,^^30^

Our study has many clinical implications. Our finding that 38% of the examined HCWs had clinically relevant anxiety symptoms and 49% of the participants had clinically relevant depression symptoms draws attention to the importance of systematically tracking the mental health of HCWs during this ongoing pandemic. The result of our study reiterates the need for periodic monitoring of the mental health status of the HCWs, especially during pandemic and provide appropriate and timely interventions, not only to improve the quality of life of HCWs, but also the quality of patient care provided. It is important that the measures taken address the key concerns of healthcare workers who are working in frontline such as adequate availability of PPE, sufficient time to spend with family, and acceptable compensations to their family in case of death.

### Limitations

There are certain limitations in our study. Firstly, our study was conducted in and around the healthcare facilities of Mangalore, a tier 2 city in India and, due to the limited geographical reach of the study, it may not be possible to generalize the results obtained. Secondly, we did not assess the mental health of the participants before the pandemic and hence their prior mental condition may act as a confounding factor in our study. Thirdly, we did not take into consideration the socio-economic parameters of the participants of our study. We recommend follow up studies to assess the progression of mental health among the HCWs after implementing workplace intervention measures for the betterment of their mental health.

The COVID-19 pandemic has had alarming implications for personal and collective health along with social and emotional functioning. The *onus* of maintaining the health of the society as well as the safety of their loved ones inserts a substantial stressor on the mental health of the frontline workers, especially the doctors and nurses in hospitals. The novel nature of the infection, inadequate testing, unknown long-term sequelae, limited availability of PPE, and extended work hours along with the other emerging concerns summate together potentially overwhelming these workers. Thus, it becomes important that the healthcare workers monitor their stress reactions, seek appropriate help both on a personal and professional level including professional mental health interventions if indicated.

## Conclusions

Our finding that 38% of the examined HCWs had clinically relevant anxiety symptoms and 49% of the participants had clinically relevant depression symptoms draws attention to the importance of systematically tracking the mental health of HCWs during this ongoing pandemic. The healthcare workers should monitor their stress reactions and seek appropriate help both on a personal and professional level. Appropriate workplace interventions including psychological support should be provided to HCWs, to ensure provision of uncompromised quality patient care.

## Data availability

### Underlying data

Open Science Framework: Covid 19 pandemic and mental health outcomes – A study among health care workers in Coastal South India. https://doi.org/10.17605/OSF.IO/J63Z5. ^33^

This project contains the following extended data:

• Data (Anonymized responses in excel sheet)
• Data key (Codes for responses)

### Extended data

Open Science Framework: Covid 19 pandemic and mental health outcomes – A study among health care workers in Coastal South India. https://doi.org/10.17605/OSF.IO/J63Z5. ^33^

This project contains the following extended data:

• Questionnaire (Blank English copy of the questionnaire used in this study).
• Information sheet and consent form.

## Reporting guidelines

Open Science Framework: STROBE checklist for ‘Covid 19 pandemic and mental health outcomes – A study among health care workers in Coastal South India’. https://doi.org/10.17605/OSF.IO/J63Z5. ^33^

Data are available under the terms of the Creative Commons Attribution 4.0 International license (CC-BY 4.0).
