# The COVID-19 pandemic and mental health outcomes – A cross-sectional study among health care workers in Coastal South India

**DOI:** 10.12688/f1000research.111193.3

**Published:** 2022-12-15

**Authors:** Rekha T, Nithin Kumar, Kausthubh Hegde, Bhaskaran Unnikrishnan, Prasanna Mithra, Ramesh Holla, Darshan Bhagawan

**Affiliations:** 1 Department of Community Medicine, Kasturba Medical College, Mangalore, Manipal Academy of Higher Education, Manipal, India

**Keywords:** Mental Health, Pandemic, Health personnel, Depression, Patient Health Questionnaire, anxiety

## Abstract

**Background: **Frontline health care workers (HCWs) are at increased risk of developing unfavourable mental health outcomes and burnout, especially during the COVID-19 pandemic. Recognizing the early warning signs of mental distress is very important to ensure the provision of quality patient care.

**Methods: **In this facility-based cross-sectional study, HCWs of the teaching hospitals affiliated to Kasturba Medical College, Mangalore were assessed regarding their mental health status using a semi-structured questionnaire. All doctors and nurses who were willing to participate from these teaching hospitals were included in the study. Data was collected over a period of four months (1 ^st^ March -30 ^th^ June 2021) till the required sample size was reached and analysed using IBM SPSS and expressed using mean (standard deviation), median (interquartile range), and proportions. Univariate analysis was done to identify the factors associated with mental health outcomes among the HCWs and the corresponding unadjusted odds ratio and 95% confidence interval were reported.

**Results: **A total of 245 HCWs [52.2% (n=128) doctors and 47.8% (n=117) nurses] were included in our study. The proportion of participants with depressive symptoms, anxiety, and insomnia assessed using PHQ-9, GAD-7, and ISI-7 scales were 49% (n=119), 38% (n=93), and 42% (n=102) respectively. Depression, anxiety, and insomnia were more likely to be experienced by HCWs aged > 27 years, females, and involved in COVID-19 patient care. (p>0.05)

**Conclusions: ** Our findings that 38% of the examined HCWs had clinically relevant anxiety symptoms and 49% had clinically relevant depression symptoms draws attention to the importance of systematically tracking the mental health of HCWs during this ongoing pandemic. HCWs should monitor their stress reactions and seek appropriate help both on a personal and professional level. Appropriate workplace interventions including psychological support should be provided to HCWs, to ensure provision of uncompromised quality patient care.

## Introduction

The coronavirus disease of 2019 (COVID-19), first identified in Wuhan, China at the end of December 2019, spread rapidly, crossing geographical boundaries and infected millions of people worldwide. The World Health Organization (WHO) acknowledged this outbreak to be of immediate public health concern and declared it a pandemic on March 11, 2020. Now, even after two years of the outbreak, and large-scale vaccination of the susceptible population, there is still no sign of an end to the pandemic. As of 10 ^th^ October 2022, WHO estimates close to 618 million confirmed cases and around 65 million deaths due to the severe acute respiratory syndrome coronavirus 2 (SARS-COV2) virus worldwide. ^1^ One of the major contributors to the global tally of COVID-19 cases, India has reported around 44 million confirmed cases and 0.52 million deaths. ^1^^,^^2^

To curb the rapid spread of COVID-19 infection, countries around the world imposed a nationwide lockdown, which was extended with rise in the number of cases reported. Once the lockdown was ceased, quarantine zones were created and region-specific restriction of movement of people was implemented. The social isolation and loss of livelihood brought about by lockdown had a huge impact on the physical and mental wellbeing of the general population.

The risk of all-cause mortality, cardiovascular diseases including hypertension, type 2 diabetes and some cancers have been linked with sedentary lifestyle. ^3^ Frequent lockdown during the pandemic resulted in the disruption of physical activity due to the closure of business, schools and recreational facilities like public parks and fitness centres, thereby forcing sedentary life style among the population. ^4^ A study in USA reported a 32% decline in physical activity during the pandemic among those who were physically active. ^5^

In addition, online classes and work from home also contributed immensely to the sedentary behaviour in general population, including children and adolescents.

The disruption of non-emergency health services during lockdown affected the routine care of population with chronic diseases like hypertension and diabetes, thereby increasing the risk of morbidities associated with the disease. ^6^ Also, those with chronic disorders were more at risk for severe COVID 19 infection. ^7^

Good mental health is essential for overall health and well-being. However, the COVID 19 pandemic has also resulted in increase in prevalence of mental health illness among the population. The economic crises due to the frequent lockdown affected the mental and psychological well-being of the population, especially among those who were unemployed and in financial debt. ^8^

Social isolation, along with the inability to see the loved ones, loss of income due to unemployment, demise of closed ones due to infection, and fear of infection are some of the triggers for mental health conditions, more so among those with pre-existing illness.

Rise in alcohol and drug use, insomnia and anxiety have also been documented during the pandemic. Covid 19 infection can itself lead to neurological and mental health complications like delirium, agitation, and stroke. During the pandemic, disruption of mental health services further compounded the already existing mental health issues. ^9^ The lack of physical activity along with poor mental health affected the overall well-being of the population during the pandemic.

Not only the general population, the pandemic affected the psychological well-being of the frontline healthcare workers (HCWs). Healthcare workers were one of the professionals who worked relentlessly during the pandemic. They were the professionals who, despite the imposed lockdown, had to work not only their own shifts of duty but sometimes work overtime or extra time to compensate for their sick colleagues.

The physical and psychological stress the HCWs experience has been documented during the past outbreaks of avian and swine influenza and other coronaviruses infection. ^10^^–^^12^ During the pandemic, the HCWs must work relentlessly and for extended hours, attending to huge caseloads and manage unforeseen medical complications. Frontline HCWs are directly involved in care of COVID patients which not only puts them at risk of acquiring infection, but also the potential spread to their family members. This fear of spreading the infection to their near and dear ones, along with unavailability of PPEs and inadequate oxygen supply, lack of standard guidelines for treatment, especially during the initial days of the pandemic and lack of psychological support have made the frontline HCWs vulnerable. ^13^

Recent literature from around the world have reported that frontline HCWs are at more risk of developing anxiety and depressive symptoms. ^14^^–^^19^ A systematic review and meta-analysis of 239 studies involving 2,71,319 subjects reported prevalence rates of anxiety and depressive symptoms of 42% and 33%, thereby providing evidence that HCWs are at increased risk of unfavourable mental health outcomes during the COVID-19 pandemic. ^20^

In India, HCWs involved in COVID-19 care were marginalized and stigmatized. HCWs in many parts of India had to perform their duties under the constant threat of aggression and violence from patient caretakers which heightened their mental distress. Refusal of entry into apartments and residences, resistance to the burial of dead bodies of HCWs, ^20^^–^^22^ and abuse of the doctors involved in screening and contact tracing were reported from different parts of the country. ^21^

Recognizing the early warning signs of mental distress is important in any population, more so among HCWs. Unfavourable mental health outcomes like anxiety, depression, insomnia, psychological distress, and burnout can affect their health, and compromise the patient safety and the quality of care provided. ^23^ Adequate interventions and coping strategies can be implemented if the mental health status of HCWs is routinely evaluated. With this background and in the context of the current COVID-19 pandemic in the district, the study was carried out to assess the mental health outcomes among the HCWs in Mangalore and the factors associated with these outcomes.

## Methods

### Ethical approval

The ethical approval was obtained from the Institutional Ethics Committee of Kasturba Medical College, Mangalore (IEC KMC MLR 05-2020/164). Electronic written informed consent was obtained from all participants on the google form. Only consenting participants were able to access the online questionnaire.

### Study area

The study was conducted in the coastal city of Mangalore, belonging to the District of Dakshina Kannada in the Southern part of India. A major commercial and educational hub, an ivory town of hospitals and medical colleges, Mangalore enjoys a high health care index ^24^ catering to patients not only from the adjoining districts, but also from the neighbouring state of Kerala.

Dakshina Kannada is among the top five highly affected districts in the State of Karnataka, by the ongoing COVID-19 pandemic. A total of 3.9 million cases have been reported till date ^25^ with Mangalore being the major contributor to the daily tally of cases.

### Participants

This facility-based cross-sectional study was carried out among the health care workers (HCWs) – doctors and nurses of the teaching hospitals affiliated to Kasturba Medical College, Mangalore.

A total of 245 HCWs were assessed for their mental health outcomes during the COVID-19 pandemic. The sample size was calculated using the formulae for cross-sectional study ^26^ design: N=4pq/d ^2^. Considering the proportion of HCWs experiencing depressive symptoms to be 50.4%, ^19^ absolute precision of 7%, power of 80%, 95% confidence interval, and a non-response error of 20%, the final sample size was calculated to be 245. Applying the population proportion to size technique, a total of 128 doctors and 117 nurses were included in the study. The doctors and nurses who were willing to participate, were included in the study, using the non-probability (convenience) sampling method, till the required sample size was reached.

### Data collection instruments

The study was conducted during the second wave of COVID-19 from 1 ^st^ March 2021 to 30 ^th^ June 2021. The information related to the study variables was collected using a semi-structured questionnaire in English which had the following sections:

• Section A: General participant information which included age, gender, designation, whether involved in COVID-19 care, and other personal details.
• Section B: Patient Health Questionnaire (PHQ-9) ^27^ consisting of 9 statements assessed the presence of depression-related symptoms among the participants. The total score ranges from 0 to 27 and is calculated by assigning each statement scores of either 0,1,2 or 3, where 0 is not at all, 1 - several days, 2 - more than half the days and 3 - nearly every day. The scores of 5, 10, and 15 represent cut points for mild, moderate and severe depression, respectively. The interpretation of the total scores obtained is as follows: Normal (0-4), mild depression (5-9), moderate depression (10-14), and severe depression (15-27).
• Section C: Generalised Anxiety Disorder (GAD-7) scale ^28^ consisting of 7 statements assessed the presence of anxiety among the participants. The total score ranges from 0 to 21 and is calculated by assigning each statement scores of either 0, 1, 2 ,or 3, where 0 is not at all, 1 - several days, 2 - more than half the days, and 3 - nearly every day. The scores of 5, 10, and 15 represent cut points for mild, moderate, and severe anxiety, respectively. The interpretation of total scores obtained is as follows: Normal (0-4), mild anxiety (5-9), moderate anxiety (10-14) and severe anxiety (15-21).
• Section D: Insomnia Severity Index (ISI) ^29^ consisting of 7 items assessed the presence of insomnia across dimensions like severity of sleep onset, sleep maintenance, and early morning awakening problems, sleep dissatisfaction, interference of sleep difficulties with daytime functioning, noticeability of sleep problems by others, and distress caused by the sleep difficulties. The statements were assigned a score of either 0,1,2,3, or 4, where 0 was no problem, 1 - mild problem, 2 - moderate problem, 3 - severe problem and 4 - very severe problem. The total score ranged from 0 to 28. The scores were interpreted as follows: Normal (0-7), subthreshold insomnia (8-14), Clinical insomnia - moderate severity (15-21) and clinical insomnia - severe (22-28).

### Reliability of the questionnaires

The Patient Health Questionnaire (PHQ-9), Generalized Anxiety Disorder (GAD-7) scale and Insomnia Severity Index (ISI) are standard questionnaires which have been used worldwide for measuring depression, anxiety and insomnia in various settings, with good reliability and internal consistency. ^27^^–^^29^ The psychometric properties of PHQ-9 and GAD-7 in Indian population were found to be comparable to western settings. ^30^ The internal reliability for PHQ-9 has been reported to be excellent with Cronbach’s α of 0.89 in primary care studies. ^31^ The GAD-7 and ISI-7 had an internal consistency score of 0.92 respectively, with good test retest reliability. ^32^^,^^33^

Pilot Testing

The questionnaire was pilot tested and validated for the content. The pilot testing was carried out in the month of February 2021 among 30 HCWs (15 doctors and 15 nurses). The participants were selected randomly using non-probability sampling. These participants were excluded from the main study. The pilot was done to evaluate the feasibility of an online survey and to finalize the questionnaire. Based on the pilot testing, questions on participants specialty, department to which they belong, and teaching experience in medical college was removed to make section A uniform for both doctors and nurses. No changes were made to Section B, section C, and section D, since they were standard questionnaires and already pre-validated.

***Data collection***

After obtaining the requisite permission from the head of the institution and concerned authorities of the hospitals, a list of doctors and nurses along with their phone numbers and email IDs working in the affiliated hospitals was obtained from the Human Resource department.

To limit personal contact with the study participants due to the prevailing pandemic, a questionnaire was prepared in Google forms ( https://www.google.co.uk/forms/) and the link was sent to the participants *via* WhatsApp or email.

The information sheet and consent form were included in the Google form. Electronic consent was obtained from each respondent on the first page of the form. Only the consenting participants were able to access the form and fill out their responses. Any personal identifiers were excluded in the form to ensure confidentiality of the participants. The google form link was circulated till the required sample size of 245 was reached.

### Data management and analysis

The collected data was extracted as a spreadsheet from Google drive and analysed using IBM SPSS (Statistical Package for Social Sciences) Statistics for Windows Version 25.0. Armonk, NY: IBM Corp). The data is expressed using mean (standard deviation), median (interquartile range), and proportions.

The interpretation of the scales used to assess the various mental health outcomes among the participants is as follows:

• General Anxiety Disorder - 7: Normal (0-4), Mild (5-9), Moderate (10-14), and severe (15-21) anxiety.
• Patient Health Questionnaire - 9: Normal (0-4), mild (5-9), moderate (10-14), and severe (15-27) depression.
• Insomnia Severity Index - 7: Normal (0-7), Subthreshold (8-14), Moderate (15-21), and Severe (22-28) insomnia.

The cut-off scores for detecting symptoms of major depression, anxiety, and insomnia were taken from the standard questionnaires. Participants were categorized to have severe symptoms if they had scored greater than the cut-off threshold.

For comparison across the groups, (gender, type of HCWs, involved in COVID-19 care) the Mann-Whitney U test was used and a ‘P’ value of <0.05 was considered statistically significant. Univariate analysis was carried out to identify the factors associated with mental health outcomes like presence of depression, anxiety, and insomnia among the HCWs and the corresponding unadjusted odds ratio and 95% confidence interval were reported.

## Results

A total of 245 HCWs, which included 52.2% (n=128) doctors and 47.8% (n=117) nurses were assessed about their mental health status in our study. The mean age of the doctors was 29.7 (±7.9) years, while that of nurses was 26.3 (± 6.2) years. A higher proportion of participants (n=66, 51.6%) among doctors were females. Majority (n=87, 68%) of the doctors and 47% (n=55) of the nurses were involved in the direct care of COVID-19 patients. The socio-demographic information of the participants is depicted in Table 1.

**Table 1.** Socio-demographic information of the study participants (N=245).

Characteristics	Doctors N=128 n (%)	Nurses N=117 n (%)
**Age group (years)**		
≤25	43 (33.6)	75 (64.1)
26-30	53 (41.4)	27 (23.1)
31-40	19 (14.8)	11 (09.4)
>40	13 (10.2)	04 (03.4)
**Gender**		
Male	62 (48.4)	-
Female	66 (51.6)	117 (100)
**Work experience (years)**		
≤10	93 (72.7)	93 (79.5)
>10	35 (27.3)	24 (20.5)
**Involved in covid care**		
Yes	87 (68.0)	55 (47.0)
No	41 (32.0)	62 (53.0)

The proportion of participants with depressive symptoms, anxiety, and insomnia assessed using PHQ-9, GAD-7 and ISI-7 scales were 49% (n=119), 38% (n=93) and 42% (n=102) respectively ( Table 2). The mental health status of the study participants is depicted in Table 3. The proportion of depression was higher among doctors (51.6%) compared to nurses (45.3%), however, this finding was not statistically significant (P>0.05). The anxiety-related symptoms were similar among doctors and nurses (doctors 39.0% vs. 36.7% in nurses). A higher proportion of nurses experienced insomnia related symptoms compared to doctors (45.2% vs. 38.2%) (P>0.05).

**Table 2.** Proportion of study participants with unfavorable mental health outcomes (N=245).

Mental health outcomes	Doctors N=128 n(%)	Nurses N=117 n(%)
Depression	66 (51.6)	53 (45.3)
Anxiety	50 (39.0)	43 (36.7)
Insomnia	49 (38.2)	53 (45.2)

**Table 3.** Mental health outcomes among the healthcare workers (HCW) (N=245).

Mental health outcomes	Category of HCW			Chi-square test (p value)
	Doctors (N=128) n (%)	Nurses (N=117) n (%)	Total (N=245) n (%)	
**Level of depression (PHQ-9)**				
Normal (0-4)	62(48.4)	64 (54.7)	126 (51.4)	1.630 (0.661)
Mild (5-9)	37 (29.0)	33 (28.2)	070 (28.6)	
Moderate (10-14)	15 (11.7)	09 (07.7)	024 (09.8)	
Severe (15-27)	14 (10.9)	11 (09.4)	025 (10.2)	
**Level of anxiety (GAD-7)**				
Normal (0-4)	78 (60.9)	74 (63.2)	152 (62.0)	5.131 * (0.159)
Mild (5-9)	27 (21.1)	28 (23.9)	055 (22.4)	
Moderate (10-14)	15 (11.7)	14 (12.0)	029 (11.8)	
Severe (15-27)	08 (06.3)	01 (00.9)	009 (03.7)	
**Level of insomnia (ISI-7)**				
Normal (0-7)	79 (61.7)	64 (54.7)	143 (58.4)	2.550 * (0.451)
Sub threshold (8-14)	36 (28.1)	38 (32.5)	74 (30.2)	
Moderate (15-21)	12 (09.4)	15 (12.8)	27 (11.0)	
Severe (22-28)	01 (00.8)	-	01 (00.4)	

* Fisher exact test.

Comparison of mental health outcome scores as assessed by the various scales is shown in Table 4. The median (IQR) scores for depression (PHQ-9), anxiety (GAD-7), and insomnia (ISI-7) for all the participants were 4.0 (1.0-8.0), 3.0 (0.5-7.0), and 6 (2-10) respectively. The median PHQ-9 and GAD-7 scores were higher among doctors compared to nurses, while nurses had a higher ISI-7 median score. However, no significant difference in mental health scores was observed across categories of HCWs, gender of the participants, and involvement in COVID-19 patients’ care (P>0.05).

**Table 4.** Comparison of mental health outcome scores among health care workers (HCW) (N=245).

Scale	Total participants	Doctors	Nurses	p value *	Male	Female	p value *	Involved in COVID-19 care		P value *
								Yes	No	
	Median (IQR)	Median (IQR)	Median (IQR)		Median (IQR)	Median (IQR)		Median (IQR)	Median (IQR)	
**PHQ-9, depression symptoms**	4 (1-8)	6.2 (2-9)	5.4 (1-8)	0.200	5.8 (1-8)	5.7 (2-9)	0.604	6.2 (1-9)	5.2 (1-8)	0.320
**GAD-7, anxiety symptoms**	3 (0.5-7)	5.0 (1-8)	3.7 (0-5.5)	0.176	4.3 (0-7)	4.5 (1-7)	0.727	4.9 (0.8-7)	4.1 (0-7)	0.495
**ISI, insomnia symptoms**	6 (2-10)	6.6 (2-10)	7.4 (2-10)	0.645	7.2 (2-10)	6.0 (2-10)	0.406	6.8 (2-10)	7.3 (2-10)	0.837

* Mann-Whitney U test.

The risk factors for developing mental health outcomes – depression, anxiety, and insomnia among HCWs is shown in Table 5.

**Table 5.** Univariate analysis showing risk factors for mental health outcomes- depression, insomnia, and anxiety among health care workers (HCW) (N=245).

	Experiencing symptoms of depression		Odds Ratio (95% CI)	P value	Experiencing symptoms of insomnia		Odds Ratio (95% CI)	P value	Experiencing symptoms of anxiety		Odds Ratio (95% CI)	P value
	Yes (N=119)	No (N=126)			Yes (N=102)	No (N=143)			Yes (N=93)	No (N=152)		
	n (%)	n (%)			n (%)	n (%)			n (%)	n (%)		
**Age group (years)**												
≤27	83 (69.7)	83 (65.9)	**1**	**0.521**	75 (73.5)	91(63.6)	**1**	**0.104**	69 (74.2)	97 (63.8)	**1**	**0.093**
>27	36 (30.3)	43 (34.1)	**1.19 (0.70-2.05)**		27 (26.5)	52 (36.4)	**1.59 (0.91-2.79)**		24 (25.8)	55 (36.2)	**1.63 (0.92-2.91)**	
**Gender**												
Male	88 (73.9)	94 (74.6)	**1**	**0.907**	25 (24.5)	105 (73.4)	**1**	**0.000001**	22 (23.7)	041 (27.0)	**1**	**0.571**
Female	31 (26.1)	32 (25.4)	**1.03 (0.58-1.84)**		77 (75.5)	038 (26.6)	**0.12 (0.06-0.21)**		71 (76.3)	111 (73.0)	**0.84 (0.46-1.52)**	
**Health care worker**												
Doctor	66 (55.5)	62 (49.2)	**1**	**0.960**	49 (48.0)	79 (55.2)	**1**	**0.269**	50 (53.8)	78 (51.3)	**1**	**0.712**
Nurse	53 (44.5)	64 (50.8)	**0.78 (0.47-1.29)**		53 (52.0)	64 (44.8)	**0.75 (0.44-1.25)**		43 (46.2)	74 (48.7)	**1.10 (0.66-1.85)**	
**Work Experience (years)**												
≤10	98 (82.4)	88 (69.8)	**1**	**0.331**	85 (83.3)	101(70.6)	**1**	**0.021**	75 (80.6)	111(73.0)	**1**	**0.175**
>10	21 (17.6)	38 (30.2)	**0.49 (0.27-0.90)**		17 (16.7)	042 (29.4)	**2.08 (1.10-3.92)**		18 (19.4)	041 (27.0)	**1.53 (0.82-2.92)**	
**Involvement in COVID care**												
Yes	72 (60.5)	70 (55.6)	**1.22 (0.73-2.04)**	**0.433**	60 (58.8)	82 (57.3)	**0.94 (0.56-1.57)**	**0.819**	54 (58.1)	88 (57.9)	**1.0 (0.58-1.67)**	**0.979**
No	47 (39.5)	56 (44.4)	**1**		42 (41.2)	61 (42.7)	**1**		39 (41.9)	64 (42.1)	**1**	

Depression was more likely to be experienced by HCWs aged >27 years (OR, 1.19; 95% CI, 0.70-2.05), female HCWs (OR, 1.03; 95% CI, 0.58-1.84), and HCWs involved in COVID-19 care (OR, 1.22; 95% CI, 0.73-2.04). Insomnia was more likely to be experienced by HCWs aged >27 years (OR, 1.59; 95% CI, 0.91-2.79) and HCWs with work experience of more than 10 years (OR, 2.08; 95% CI, 1.10-3.92). Anxiety was more likely to be experienced by HCWs aged >27 years (OR, 1.63; 95% CI, 0.92-2.91), nurses (OR, 1.10; 95% CI, 0.66-1.85), and HCWs with work experience of more than 10 years (OR, 1.53; 95% CI, 0.82-2.92). However, none of these factors was significantly associated with mental health outcomes (P>0.05), except HCWs with work experience of more than 10 years, which was significantly associated with experiencing insomnia symptoms (P<0.05).

## Discussion

Health-care workers (HCWs) have been at the forefront since the beginning of the pandemic providing uncompromising care to those infected with COVID-19. However, the extended period of duty hours and the constant fear of getting infected or spreading the infection to family members has negatively impacted their physical and mental health. Unfavourable mental health outcomes – depression, insomnia and anxiety were also reported among our study participants.

The prevalence of depression was 51.5% in our study. Anxiety was reported among 38%, while insomnia was present in 42% of the participants. The prevalence of unfavourable mental health outcomes in our study is high compared to a similar study from another part of India where depression was reported among 47.4% of the HCWs, while anxiety and insomnia were seen among 29.0% and 32.2% of the participants respectively. ^34^ Similar studies conducted among the HCWs during the pandemic from different parts of the world have reported a prevalence of depression ranging from 8.9% to 77.2%, ^14^^–^^19^^,^^23^^,^^35^^–^^45^ anxiety 14.5% to 88%, ^14^^–^^19^^,^^36^^–^^40^^,^^48^^–^^52^ and insomnia 9.9% to 85.4%. ^37^^,^^38^^,^^40^^–^^51^

Many factors can contribute to unfavorable mental health outcomes among HCWs. The long duty hours and overflowing outpatient departments (OPDs), along with acute shortage of trained staff and personal protective equipment (PPE), fear of contracting the infection and spreading the disease to their family members, and continuous performance evaluation results in psychological distress in most HCWs, ultimately leading to burnout. ^37^^,^^52^^–^^57^ HCWs are also faced with several decision-making dilemmas during a pandemic including allocation of resources, care for a severely ill/dying patient, and aligning patient needs with those of family members further resulting in mental distress. ^58^^,^^59^ All this is compounded by a prolonged period of separation from family members during the pandemic and/or a lack of any other form of support system. ^10^^–^^13^^,^^33^^,^^34^

Studies from different parts of the world have reported a variety of factors contributing to unfavourable mental health outcomes. A study from the Eastern Mediterranean region reported that the presence of a pre-existing mental illness, being isolated for COVID-19, and having children was significantly associated with experiencing depressive symptoms ^43^ while insomnia was significantly associated with HCWs working in an isolation unit in a study in China. ^41^ The absence of psychological support at the workplace as a factor for experiencing poor mental outcomes was reported in studies conducted in China and Albania, ^41^^,^^44^ while fear of getting infected and transmitting COVID-19 was associated with experiencing depressive symptoms among HCWs in Switzerland and China. ^40^^,^^41^

The frontline HCWs being directly involved in the examination, diagnosis, and treatment of COVID-19 makes them more vulnerable to contracting the infection. The constant fear of being at risk of getting infected may lead to psychological distress and burnout among them. Several studies have reported that the HCWs involved in direct care for COVID-19 patients were found to have unfavourable mental health outcomes. ^20^^,^^37^^,^^42^^,^^45^

In our study, unfavourable mental health outcomes – depression, anxiety and insomnia were observed among HCWs >27 years of age, nurses, and those having work experience of more than 10 years. Similar observations were reported in various studies among HCWs around the world.

A higher proportion of doctors and all the nurses in our study were females. Females were found to have a higher risk of experiencing poor mental outcomes in studies conducted in Asia and Africa ^19^^,^^27^^,^^35^^,^^38^^,^^50^ whereas another study from Africa reported male HCWs and physicians to be more at risk for experiencing distress. ^45^^,^^46^ In general, female HCWS, especially nurses, were found to be more likely to experience depression and insomnia related symptoms. ^27^^,^^35^^,^^38^^,^^40^^,^^41^^,^^50^ This vulnerability of nurses may be attributed to longer duration of time spent in patient care compared to doctors, along with increased work load and frequent night shifts. ^38^ Studies have shown that nurses were more likely to have job uncertainty and financial concerns, which along with fear of getting infected due to prolonged time spent in the ward, could have compounded the anxiety and insomnia experienced by them. ^60^^,^^61^

In our study, work experience of more than 10 years was found to be significantly associated with experiencing insomnia symptoms. A similar study from Nepal reported that HCWs having less than 5 years’ work experience were less likely to experience insomnia related symptoms, compared to those with more than 5 years of experience. This is in contrast to studies from Turkey ^62^ and China ^63^ where having less work experience was significantly associated with unfavourable mental health outcomes. The most likely explanation for this observation would be that the HCWs with more than 10 years’ experience would be married and have a family, which would make them anxious due to fear of spreading the infection to their family. Also, during the pandemic, the more experienced HCWs might have been given more responsibilities as compared to the newer staff.

A higher age usually corresponds to more experience in handling complex situations and prompt decision making especially during the pandemic, when the health system was overburdened. ^38^ However, in our study HCWs above 27 years of age were more likely to experience depression, anxiety and insomnia. A study in China reported that the HCWs in the 31-40 years of age were worried about infecting their family, while those above 40 years of age were stressed due to the concern regarding their own safety during the pandemic. ^60^ In contrast, younger HCWs in the age group between 21-30 years were found to experience unfavourable mental health outcomes in studies from Italy, ^37^ Finland ^46^ and Hong Kong. ^50^

Our study has many clinical implications. The presence of clinically relevant anxiety and depressive symptoms among 38% and 49% HCWs respectively in our study draws attention to the importance of regular and systematic tracking of mental health status of HCWs during this ongoing pandemic. The result of our study reiterates the need for periodic monitoring of the mental health status of the HCWs, especially during pandemic and provide appropriate and timely interventions, not only to improve the quality of life of HCWs, but also the quality of patient care provided. It is important that the measures taken address the key concerns of healthcare workers who are working in frontline such as adequate availability of PPE, sufficient time to spend with family, and acceptable compensations to their family in case of death.

### Limitations

There are certain limitations in our study. Firstly, our study was conducted in and around the healthcare facilities of Mangalore, a tier 2 city in India and, due to the limited geographical reach of the study, it may not be possible to generalize the results obtained. Secondly, we did not assess the mental health of the participants before the pandemic and hence their prior mental condition may act as a confounding factor in our study. Thirdly, we did not take into consideration the socio-economic parameters of the participants of our study. We recommend follow up studies to assess the progression of mental health among the HCWs after implementing workplace intervention measures for the betterment of their mental health.

The COVID-19 pandemic has had alarming implications for personal and collective health along with social and emotional functioning. The *onus* of maintaining the health of the society as well as the safety of their loved ones inserts a substantial stressor on the mental health of the frontline workers, especially the doctors and nurses in hospitals. The novel nature of the infection, inadequate testing, unknown long-term sequelae, limited availability of PPE, and extended work hours along with the other emerging concerns summate together potentially overwhelming these workers. Thus, it becomes important that the healthcare workers monitor their stress reactions, seek appropriate help both on a personal and professional level including professional mental health interventions if indicated.

## Conclusions

A higher proportion of doctors compared to nurses had symptoms of depression and insomnia, while the proportion of doctors and nurses experiencing anxiety related symptoms is almost similar. Overall 49% of the HCWs had clinically relevant depressive symptoms and 38% had clinically relevant anxiety symptoms. This draws attention to the importance of systematically tracking the mental health of HCWs during this ongoing pandemic. The healthcare workers should monitor their stress reactions and seek appropriate help both on a personal and professional level. Appropriate workplace interventions including psychological support should be provided to HCWs, to ensure a sound mental health, so as to provide uncompromised quality health care to the patients.

## Data availability

### Underlying data

Open Science Framework: Covid 19 pandemic and mental health outcomes – A study among health care workers in Coastal South India. https://doi.org/10.17605/OSF.IO/MU9E8. ^64^

This project contains the following extended data:

• Data (Anonymized responses in excel sheet)
• Data key (Codes for responses)

### Extended data

Open Science Framework: Covid 19 pandemic and mental health outcomes – A study among health care workers in Coastal South India. https://doi.org/10.17605/OSF.IO/MU9E8. ^64^

This project contains the following extended data:

• Questionnaire (Blank English copy of the questionnaire used in this study).
• Information sheet and consent form.

## Reporting guidelines

Open Science Framework: STROBE checklist for ‘Covid 19 pandemic and mental health outcomes – A study among health care workers in Coastal South India’. https://doi.org/10.17605/OSF.IO/MU9E8. ^42^

Data are available under the terms of the Creative Commons Attribution 4.0 International license (CC-BY 4.0).
