# Supplementary material for: The COVID-19 pandemic and mental health outcomes – A cross-sectional study among health care workers in Coastal South India
Source: F1000Res. 2023 Feb 20;11:676. Originally published 2022 Jun 20. [Version 4] doi: 10.12688/f1000research.111193.4 (PMC10186062; doi:10.12688/f1000research.111193.4)
Supplement: Peer review history

# Peer review: Reviewer response for version 4

**DOI:** 10.5256/f1000research.144474.r163944

**Published:** 2023-05-15

**Authors:** Devi Wulandari

**Affiliations:** 1 Department of Psychology, Paramadina University, Jakarta, Indonesia

There are several results that need to be addressed in the discussion section i.e: 

1. No significant differences between doctors and nurses in terms of depression, anxiety, and insomnia
2. Age and  involvement  in COVID-19 care were not significantly associated with mental health outcome 

Is the work clearly and accurately presented and does it cite the current literature?

Partly

If applicable, is the statistical analysis and its interpretation appropriate?

No

Are all the source data underlying the results available to ensure full reproducibility?

Yes

Is the study design appropriate and is the work technically sound?

Partly

Are the conclusions drawn adequately supported by the results?

Partly

Are sufficient details of methods and analysis provided to allow replication by others?

Partly

Reviewer Expertise:

Health psychology

I confirm that I have read this submission and believe that I have an appropriate level of expertise to confirm that it is of an acceptable scientific standard.

# Peer review: Reviewer response for version 3

**DOI:** 10.5256/f1000research.141822.r158225

**Published:** 2023-01-30

**Authors:** Devi Wulandari

**Affiliations:** 1 Department of Psychology, Paramadina University, Jakarta, Indonesia

Thank you for your latest revision. There are some minor feedback: 

 Results section:

1. 2nd paragraph: since the P values of the analysis is higher than 0.05 you cannot conclude that nurses experience more insomnia compared to doctors. 
2. last paragraph: Gender was significantly associated with mental health outcome (insomia), should be mentioned in the results section and discussed

Is the work clearly and accurately presented and does it cite the current literature?

Partly

If applicable, is the statistical analysis and its interpretation appropriate?

No

Are all the source data underlying the results available to ensure full reproducibility?

Yes

Is the study design appropriate and is the work technically sound?

Partly

Are the conclusions drawn adequately supported by the results?

Partly

Are sufficient details of methods and analysis provided to allow replication by others?

Partly

Reviewer Expertise:

Health psychology

I confirm that I have read this submission and believe that I have an appropriate level of expertise to confirm that it is of an acceptable scientific standard, however I have significant reservations, as outlined above.

# Response

**Published:** 2023-02-15

**Authors:** Nithin Kumar

**Affiliations:** Kasturba Medical College Hospital, India

Thank you for your suggestion. Kindly find the response to your comments.

 Results section:

1. 2nd paragraph: since the P values of the analysis is higher than 0.05 you cannot conclude that nurses experience more insomnia compared to doctors. 

Thank you for the suggestion. We have added a line to the results clearly stating that the observation that nurses had a higher proportion of insomnia was not statistically significant.

  

1. Last paragraph: Gender was significantly associated with mental health outcomes (insomnia), which should be mentioned in the results section and discussed.

Thank you. We have incorporated the same in the result section and have added in the discussion.

# Peer review: Reviewer response for version 2

**DOI:** 10.5256/f1000research.139956.r154705

**Published:** 2022-11-22

**Authors:** Devi Wulandari

**Affiliations:** 1 Department of Psychology, Paramadina University, Jakarta, Indonesia

Thank you very much for your revision. Overall, the present article has shown significant improvements. There are several additional feedbacks to increase the article's credibility. 

1. The 2nd paragraph in the introduction: please add references regarding lockdown impacts on physical, mental and well-being in the population 
2. Data collection instruments: please add information regarding the calculation of participants score and reliability score for each instruments in the study 
3. in the result section, please add: a demographic table for the research participants before table 1 to give an overall description 
4. Information regarding 51.5% prevalence of depression in the study should be informed in a table for result sections
5. Paragraph 3 in the discussion should be added with more references 
6. Since only work experiences of more than 10 years was the only significant risk factors, the discussion should only discuss the association of work experience with mental health outcomes. 
7. Please also discuss the explanation regarding factors that were not statistically significance 

Is the work clearly and accurately presented and does it cite the current literature?

Partly

If applicable, is the statistical analysis and its interpretation appropriate?

No

Are all the source data underlying the results available to ensure full reproducibility?

Yes

Is the study design appropriate and is the work technically sound?

Partly

Are the conclusions drawn adequately supported by the results?

Partly

Are sufficient details of methods and analysis provided to allow replication by others?

Partly

Reviewer Expertise:

Health psychology

I confirm that I have read this submission and believe that I have an appropriate level of expertise to confirm that it is of an acceptable scientific standard, however I have significant reservations, as outlined above.

# Response

**Published:** 2022-12-03

**Authors:** Nithin Kumar

**Affiliations:** Kasturba Medical College Hospital, India

Thank you sir for your valuable comments. We have modified the article as per your suggestions.

  

1. **The 2nd paragraph in the introduction: please add references regarding lockdown impacts on physical, mental and well-being in the population.**
**Response:** Thank you, sir. We have updated paragraph 2 of the introduction with the impact of the lockdown on the physical and mental well-being of the population. The reference list has been updated accordingly.
2. **Data collection instruments: Please add information regarding the calculation of participants score and reliability score for each instruments in the study.**
**Response: **Thank you, sir. We have provided the information on the calculation of participants' scores. However, we did not calculate the reliability scores for the questionnaires, since they were standard questionnaires. However, we have provided references depicting the reliability of the questionnaires, in Indian and western settings.
3. **In the result section, please add: a demographic table for the research participants before table 1 to give an overall description.**
**Response: **Thank You, sir. We have incorporated a table on demographic details in the manuscript as per your suggestion.
4. **Information regarding 51.5% prevalence of depression in the study should be informed in a table for result sections.**
**Response:** Thank you, sir. We have incorporated a new table depicting the proportion of doctors and nurses with unfavorable mental health outcomes.
5. **Paragraph 3 in the discussion should be added with more references.**
**Response**: Thank you sir. We have supported the paragraph 3 of discussion with new references.
6. **Since only work experiences of more than 10 years were the only significant risk factors, the discussion should only discuss the association of work experience with mental health outcomes. **
**Response: **Thank you, sir. We have discussed the factors found to be significant in our study.
7. **Please also discuss the explanation regarding factors that were not statistically significance.**
**Response:** Thank you sir. We have incorporated explanations for factors like gender, and HCWs which were not found to be significant in our study.

# Peer review: Reviewer response for version 2

**DOI:** 10.5256/f1000research.139956.r154706

**Published:** 2022-11-07

**Authors:** Sunil Kumar Raina

**Affiliations:** 1 Department of Community Medicine, Dr. RP Govt. Medical College, Tanda, Himachal Pradesh, India

The manuscript is approved for indexing.

Is the work clearly and accurately presented and does it cite the current literature?

Yes

If applicable, is the statistical analysis and its interpretation appropriate?

Yes

Are all the source data underlying the results available to ensure full reproducibility?

Yes

Is the study design appropriate and is the work technically sound?

Yes

Are the conclusions drawn adequately supported by the results?

Yes

Are sufficient details of methods and analysis provided to allow replication by others?

Yes

Reviewer Expertise:

Epidemiology

I confirm that I have read this submission and believe that I have an appropriate level of expertise to confirm that it is of an acceptable scientific standard.

# Peer review: Reviewer response for version 1

**DOI:** 10.5256/f1000research.122887.r149463

**Published:** 2022-10-03

**Authors:** Devi Wulandari

**Affiliations:** 1 Department of Psychology, Paramadina University, Jakarta, Indonesia

1. The work was clearly presented however the study cited 72% of current literature. Adding more current literature, especially in the introduction section will suffice.
2. Appropriateness of study design. There is some information that needs to be completed in the method section, such as criteria for the cutoff score and information regarding the reliability of the measurement.
3. Statistical analysis. There are some questionable interpretations such as the proportion of participants with depressive symptoms higher among doctors but in fact the result (chi square) was not significant.
4. Conclusion. Since there are several questionable results from the study, thus the conclusion were not adequately supported. 

Is the work clearly and accurately presented and does it cite the current literature?

Partly

If applicable, is the statistical analysis and its interpretation appropriate?

No

Are all the source data underlying the results available to ensure full reproducibility?

Yes

Is the study design appropriate and is the work technically sound?

Partly

Are the conclusions drawn adequately supported by the results?

Partly

Are sufficient details of methods and analysis provided to allow replication by others?

Partly

Reviewer Expertise:

Health psychology

I confirm that I have read this submission and believe that I have an appropriate level of expertise to confirm that it is of an acceptable scientific standard, however I have significant reservations, as outlined above.

# Response

**Published:** 2022-10-17

**Authors:** Nithin Kumar

**Affiliations:** Kasturba Medical College Hospital, India

1. The work was clearly presented however the study cited 72% of current literature. Adding more current literature, especially in the introduction section will suffice.
**Response:** Thank you sir. We have incorporated current literature in the introduction and discussion as per your suggestion.  
2. Appropriateness of study design. There is some information that needs to be completed in the method section, such as criteria for the cut off score and information regarding the reliability of the measurement.
**Response:** Thank you sir. Since the scales used to assess depression, anxiety and insomnia in our study was standard, cut-offs scores mentioned by the scales were used. A note on reliability of the scales have been added in the methods section. The cut-off scores used are also mentioned in the methods section.  
3. Statistical analysis. There are some questionable interpretations such as the proportion of participants with depressive symptoms higher among doctors but in fact the result (chi square) was not significant.
**Response: **Thank you sir for pointing out the error. We have modified the statement and added the P value so that the interpretation is clear.
4. Conclusion. Since there are several questionable results from the study, thus the conclusion were not adequately supported. 
**Response:** The conclusion has been re-written according to the results.

# Peer review: Reviewer response for version 1

**DOI:** 10.5256/f1000research.122887.r142248

**Published:** 2022-07-19

**Authors:** Sunil Kumar Raina

**Affiliations:** 1 Department of Community Medicine, Dr. RP Govt. Medical College, Tanda, Himachal Pradesh, India

The article is relevant in the context of the prevailing COVID 19 pandemic and the taxing effect it has, not only on the physical health of the HCWs, but also their mental health.

 The manuscript is well written. However, there are few queries I would like the author to address in the manuscript.

- Has the questionnaire used in the study been validated?
- Was the questionnaire translated to the local vernacular language for the nurses?
- How was the data collected?
- For how many health care professionals' questionnaires were distributed in order to reach the required sample size? What was the non-response rate?
- How can you attribute the present mental health status to COVID 19? Can it be due to existing mental health condition or health status prior to COVID 19 pandemic ? Did you gather information regarding past mental health status?

Is the work clearly and accurately presented and does it cite the current literature?

Yes

If applicable, is the statistical analysis and its interpretation appropriate?

Yes

Are all the source data underlying the results available to ensure full reproducibility?

Yes

Is the study design appropriate and is the work technically sound?

Yes

Are the conclusions drawn adequately supported by the results?

Yes

Are sufficient details of methods and analysis provided to allow replication by others?

Yes

Reviewer Expertise:

Epidemiology

I confirm that I have read this submission and believe that I have an appropriate level of expertise to confirm that it is of an acceptable scientific standard, however I have significant reservations, as outlined above.

# Response

**Published:** 2022-07-28

**Authors:** Nithin Kumar

**Affiliations:** Kasturba Medical College Hospital, India

Thank you for your review of our paper titled The COVID-19 pandemic and mental health outcomes – A cross-sectional study among health care workers in Coastal South India"

 Kindly find our reply for your queries below:

1. **Has the questionnaire used in the study been validated?**
  The Patient Health Questionnaire (PHQ-9), Generalized Anxiety Disorder (GAD-7) scale, and Insomnia Severity Index (ISI) -all are standard questionnaires which are pre-validated.
  However, a pilot testing was carried out among 30 HCWs (15 doctors and 15 nurses), to check the usability/feasibility of the questionnaire.
  Based on the pilot testing, changes were made to Section A (General participant information)-like income of the participants and permanent addresses were removed.
  No changes were made to Section B (PHQ-9), Section C (GAD-7) and ISI (Section D).
2. **Was the questionnaire translated to the local vernacular language for the nurses?**
  The questionnaires were not translated into the local vernacular language. Pre-validated standard questionnaire in English language was sent to all the study participants – doctors and nurses via google form link. All the nurses were proficient in English language.
3. **How was the data collected?**
  To limit the personal contact with the study participants due to the prevailing pandemic, the questionnaire was prepared in Google forms and the link was sent to the participants via WhatsApp or email.
4. **For how many health care professionals' questionnaires were distributed in order to reach the required sample size? What was the non-response rate?**
  To reach the sample size of 245 (128 doctors and 117 nurses), the link was sent to 280 health care workers, which included 150 doctors and 130 nurses.
  The response rate for doctors was 85.3% and 90.3% for nurses.
5.  
**How can you attribute the present mental health status to COVID 19? Can it be due to existing mental health condition or health status prior to COVID 19 pandemic? Did you gather information regarding past mental health status?**
  Yes. We did not take into consideration the existing mental health condition of the health care professionals and that is the limitation of our study.
